# Morphological crypsis within a crustacean species complex is driven by within-species phenotypic diversification

**DOI:** 10.1038/s41598-025-29270-1

**Published:** 2025-12-02

**Authors:** Jana Kabus, Carolin Schaub, Dennis Fritsche, Berardino Cocchiararo, Cene Fišer, Michał Grabowski, Ioannis Karaouzas, Spase Shumka, Jonas Jourdan

**Affiliations:** 1 Department Aquatic Ecotoxicology, Goethe University of Frankfurt, Max-von-Laue-Straße 13, 60438 Frankfurt am Main, Germany; 2 Conservation Genetics Section, Senckenberg Research Institute, Senckenberg Gesellschaft für Naturforschung, Senckenberganlage 25, 60325 Frankfurt/Main, Germany; 3 Biotechnical Faculty, University of Ljubljana, Jamnikarjeva 101, 1000 Ljubljana, Slovenia; 4 Department of Invertebrate Zoology and Hydrobiology, Faculty of Biology and Environmental Protection, University of Łódź, Banacha 12/16, 90-237 Łódź, Poland; 5 Hellenic Centre for Marine Research, Institute of Marine Biological Resources and Inland Waters, 46.7km Athens-Sounio Av, 19013 Anavyssos, Greece; 6 Faculty of Biotechnology and Food, Agricultural University of Tirana, Tirana, Albania

**Keywords:** Amphipod, Convergent evolution, Cryptic species, Molecular taxonomy, Phenotypic Plasticity, Phylogenetic niche conservatism, Biodiversity, Ecological genetics, Evolutionary ecology, Freshwater ecology

## Abstract

Cryptic species complexes-genetically divergent lineages with similar phenotypes may appear cryptic not due to true similarity, but because high within-species morphological variation masks interspecies differences, particularly in heterogeneous environments. This study tests whether such lineages exhibit morphological convergence across similar environmental gradients or context-dependent divergence, challenging their cryptic status. We propose a theoretical framework to assess phenotypic differentiation and apply it using the amphipod complex *Gammarus roeselii*, which diversified in the Balkan Peninsula before one lineage expanded into Central Europe. We examined five lineages across similar environmental gradients to evaluate their phenotypic divergence. Our findings confirmed their cryptic status. However, phenotypic differentiation along environmental gradients revealed MOTU-specific adaptive trajectories: traits under strong selection (e.g., anti-predator defences) showed convergent responses, while morphological (e.g., body length) and physiological traits (e.g., gill area) diverged, likely due to local selection. Some MOTUs exhibited distinct, partially divergent sexual dimorphism, suggesting varied reproductive or ecological strategies within cryptic lineages. Phenotypic variation was greater within than between MOTUs, reinforcing their cryptic status and revealing strong local adaptive responses. This complex exemplifies how adaptive divergence and biogeographic history jointly shape biodiversity, with morphological stasis despite genetic and ecological differentiation exposing hidden speciation and environmental responses.

**Supplementary Information:**

The online version contains supplementary material available at 10.1038/s41598-025-29270-1.

## Introduction

In the vast majority of cases, related species change their phenotypic appearance over time and differentiate from each other due to evolutionary processes such as natural selection, genetic drift, mutation and sexual selection. Diverse environmental pressures lead to adaptations that drive phenotypic divergence, while random changes and mating preferences also contribute to phenotype variation^1,2^. These processes result in related species evolving different phenotypes to better survive and reproduce in their respective environments. In some cases, however, species remain identical in their phenotypes despite phylogenetic differentiation^3–5^. We refer to these species as cryptic species, which cannot be distinguished morphologically from one another despite many millions of years of independent evolutionary history. The similarity in morphology of cryptic species may be maintained by a stabilising selection, as they occupy similar habitat and use of the same resources^6–8^.

Cryptic species posing significant challenges to taxonomy and biodiversity research, which historically relied on observable traits like morphology, coloration, or size to classify organisms. This can lead to misidentification, underestimation of biodiversity and errors in ecological studies, as researchers may unwittingly group several genetically distinct lineages under a single species name^8–11^. During the last decades, cryptic species were frequently detected as a by-product in phylogeographic studies^8^. In these studies, the genetically distinct lineages are provisionally classified into molecular operational taxonomic units (MOTUs) based on specific thresholds of genetic divergence based on DNA sequences. Although it is still difficult to establish a fixed species threshold^12,13^, the use of COI barcoding as species identification has become a useful tool. With the low-cost identification of cryptic species complexes now possible, this hidden aspect of biodiversity has the potential to provide profound insights into evolutionary processes and ecological adaptations^14,15^.

**Fig. 1:**
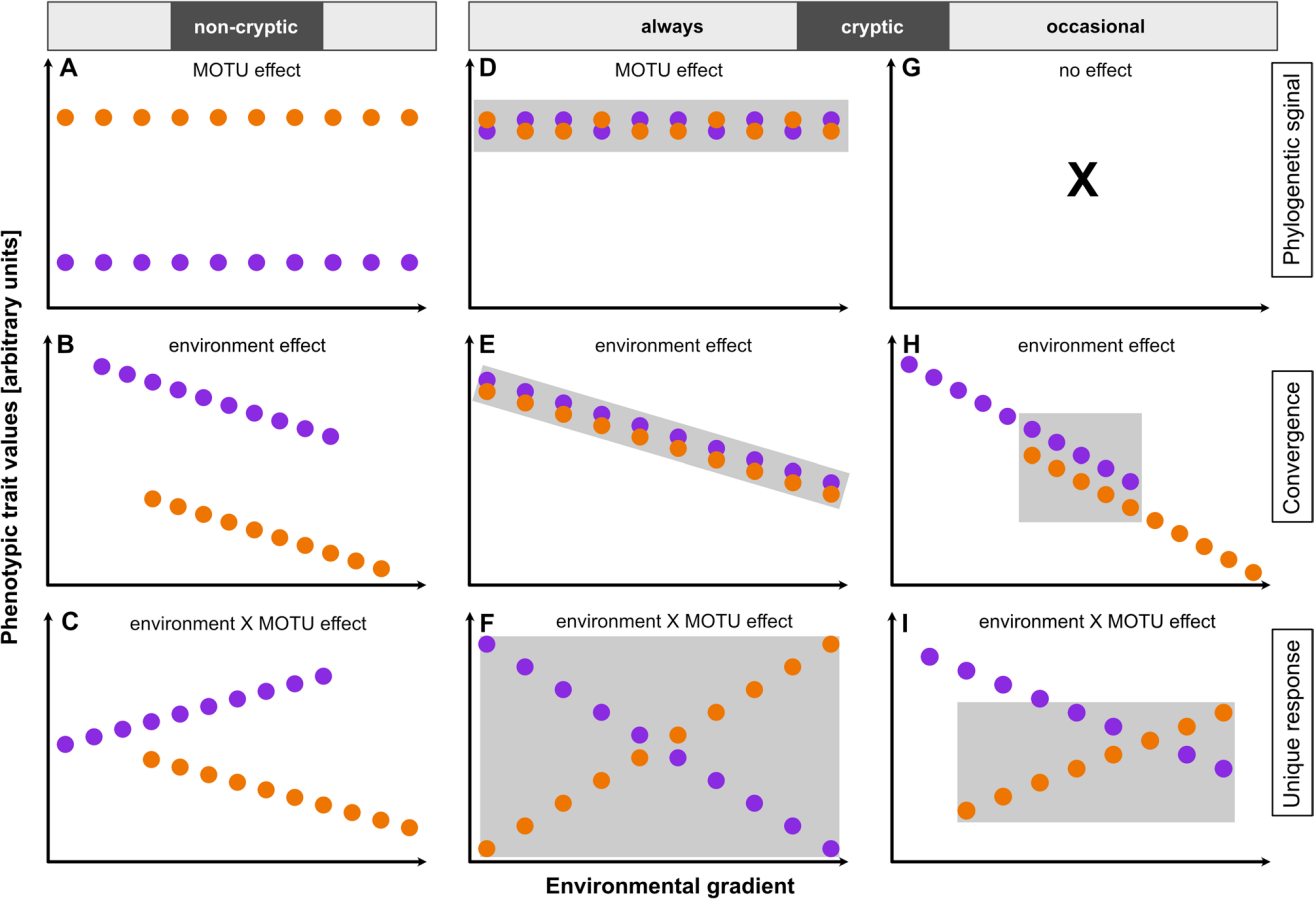
Illustration of hypothetical effects of shared and unique phenotypic trait divergence along an environmental gradient in two genetically identified species (molecular operational taxonomic units; MOTUs; purple and orange). (**a**–**c**) Both MOTUs occur across replicated environmental gradients, but their phenotypic traits never overlap. In these cases we clearly have morphologically differentiated MOTUs (i.e., non-cryptic species). (**a**) Unique phenotypes could arise from different evolutionary histories of both MOTUs (i.e., represent a phylogenetic signal) and are independent of the environmental gradient. (**b**) Selective forces could result in convergent (shared) patterns of divergence in both MOTUs, showing similar tendencies of phenotypic differentiation that can still be distinguished. (**c**) Phenotypic diversification could also be due to unique (MOTU-specific) responses to environmental conditions. (**d**–**f**) Both MOTUs can be cryptic irrespective of environmental conditions. This may be due to (**d**) an identical phenotypes that arise independent of the environmental gradient, or both MOTUs show (**e**) the same or (**f**) the opposite trait divergence across the environmental gradient. In the two latter cases, the trait divergence would be more pronounced within than between MOTUs. (**g**–**i**) Moreover, it could also be that both MOTUs can be distinguished most of the time and are cryptic only under certain environmental conditions (i.e., they can occasionally be cryptic). (**h**) This might be the case, when both MOTUs show similar phenotypic trait divergence with overlapping traits under some conditions, while they differ under other circumstances. (**i**) A phylogenetic signal could also result in different phenotypes that respond in the opposite direction across the environmental gradient. Shaded areas represent cryptic phenotypes. Text in the graphs indicate significant effects of the main factor (‘MOTU’), the covariate (‘environmental gradient’), or their interaction in analyses of covariance [(M)ANCOVA] using phenotypic trait values as the dependent variable.

The evolutionary mechanisms underlying morphological similarity among species are poorly understood^3,8^. Technically, a pair of species cannot be told apart when within-species variation exceeds the between-species variation. Hypotheses explaining morphological similarity commonly invoked similar selective pressures, driving mean values of quantitative traits towards the same adaptive optima^3,8^. However, virtually completely neglected remained mechanisms that increase within-species variation in comparison to between-species differences. Indeed, phenotypic variation within species (i.e., between populations across a species’ range) is quite common. Species that live in spatially or temporary heterogeneous environment experience diverse selective pressure, which both, increase and maintain phenotypic variation^16^. If so, morphological crypsis due to increased phenotypic variation should be common among species living in heterogeneous environment or across environmental gradients (Fig. 1). Populations occurring across environmental gradients face various forms of both natural and sexual selection^17–20^. For example, environmental gradients in rivers are made up by an array of selective forces that vary from source to downstream regions, and local variation in fitness maxima can result in gradual spatio-temporal variation of phenotypic traits^2,17^. In this study, we fill the knowledge gap and test the hypothesis that species living along the environmental gradients increase their phenotype variation and consequently cannot be told apart on a basis of morphological variation.

We studied phenotype variation of morphological traits associated with life-history traits^21,22^ of the amphipod species complex *Gammarus roeselii* Gervais, 1835. These traits are shaped by evolutionary pressures and can vary widely among species and populations in response to their specific environment^23,24^. Key life-history traits include morphological and reproductive features which can have interdependencies due to physiological constraints^18,25–27^.

The amphipod *Gammarus roeselii* Gervais, 1835 is a prominent example of a cryptic species complex, widely distributed across Europe^15,28^. This species complex consists of at least 13 MOTUs, with the earliest clades likely diverging in the early Miocene, approximately 18 million years ago^28^. The species complex shows its greatest diversification on the Balkan Peninsula – a known hotspot for biodiversity^15,28^. The complex geological and climatic history of the Balkan peninsula likely contributes to the genetic differentiation observed^28^. One of the genetic lineages (MOTU C) has also spread to central Europe and has been increasingly studied in recent years, partly because they often colonize heavily anthropogenically shaped rivers^29–33^. However, recent studies have shown that the other MOTUs of the *G. roeselii* complex also colonize anthropogenically polluted waters on the Balkan Peninsula and are comparable in their tolerance to pollutants, despite being geographically much less widespread^34,35^. Phenotypic differentiation along environmental gradients has already been demonstrated for MOTU C. For example, gradual changes in gill size and reproductive traits were observed, likely reflecting adaptive responses to pollution and resource availability^30,32^.

Specifically, our work aims to quantitatively characterize the cryptic species complex *G. roeselii* for the first time using selected phenotypic traits to underline the cryptic status and that none of the selected life-history traits clearly differ between the genetic lineages. We then investigate the extent to which the genetic lineages phenotypically differentiate along environmental gradients following a theoretical framework of different hypothetical differentiations (Fig. 1). We hypothesize that (i) convergent differentiation has contributed to the formation of the cryptic species complex and (ii) phenotypic trait differentiation within MOTUs exceeds the divergence observed between MOTUs, suggesting that genetic thresholds for species delimitation may not align with phenotypic trait boundaries.

## Materials and methods

### Sampling

**Fig. 2:**
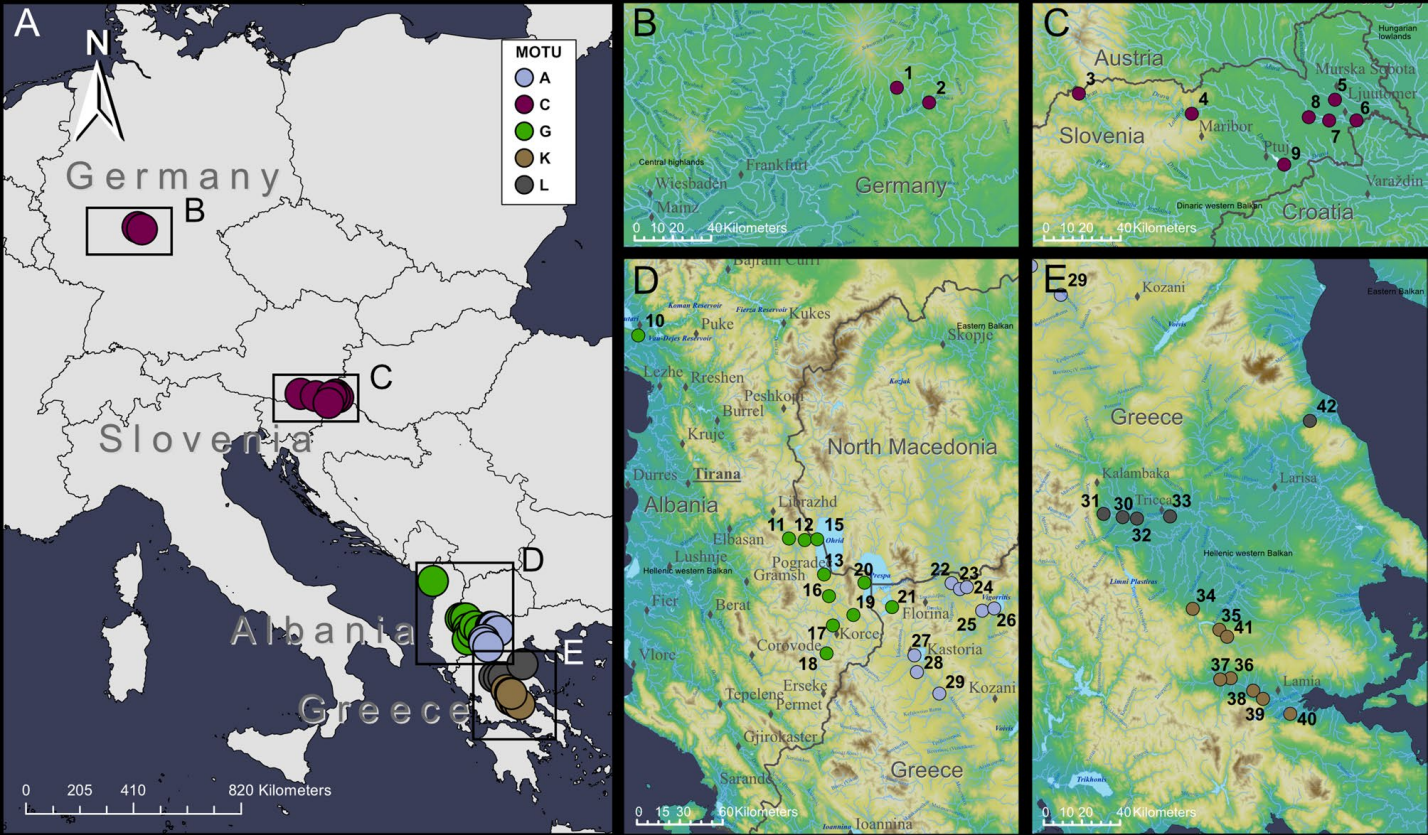
Map of all *G. roeselii* sites covering the diversification hotspot on the Balkan Peninsula, as well as occurrences of the widespread MOTU C in Slovenia and central Germany. (**A**) Overall map of Europe with regions of sampling. (**B**) Two sampling sites of MOTU C within the Main river catchment. (**C**) Sampling sites in Slovenia of MOTU (**C**, **D**) Sampling sites of MOTU G in Albania and MOTU A in northern Greece. (**E**) Sampling sites of MOTU L and K in southern Greece. Maps were created using ArcGIS Desktop v.10.8.

In order to investigate phenotypic differentiation within *G. roeselii* species complex, we sampled several sites initially studied by Grabowski et al.^28^. To ensure sufficient occurrences across varying environmental conditions, we supplemented these sampling site with additional ones, so that we sampled a total of 35 sites (Fig. 2). The numbering of these sites are in accordance to Kabus et al.^34^. Coordinates of the sampling sites are given in Supplementary Table S1. The sampling campaign was conducted in September 2021 in Germany, Slovenia, Albania and Greece. We conducted multi-habitat kick-sampling using hand nets (Bioform V2A; mesh size 500 μm), covering the main substrate types present at each site, including coarse and fine gravel, sand, leaf litter, woody debris, and bryophytes, to ensure representative sampling across microhabitats. Captured animals were immediately preserved in individual 2 ml microcentrifuge tubes filled with 96% ethanol. Individuals in the centrifuge tubes were stored at 10 °C until processed in the laboratory for molecular analysis. From each site, 70 individuals were collected, of which at least 20 sexually mature males and females were randomly selected in the laboratory for further analysis; juveniles identified as immature were excluded. Care was taken to ensure that the selection process was randomized to avoid introducing size bias. The species do not have any protected status. Permission was granted by the Hellenic Ministry of Environment and Energy (Permission No: ΥΠΕΝ/ΔΔΔ/7316/280) and the Albanian National Agency of Protected Areas (Permission No: 194, issued on 16.02.2021).

### Phenotypic characterization

#### General traits

The life history analysis considered morphological and reproductive traits that are influenced by abiotic and biotic factors. These traits are subject to natural and sexual selection. The measurements were carried out by using the methods described by Jourdan et al.^30^ that provided a detailed measuring description.

The specimens fixed in 96% ethanol were firstly sexed according to external features. We phenotypically characterized a total of 1,247 individuals. Although we tried to measure 20 individuals per sex per each sampled site, this was not always possible. Therefore we ended up with 673 males and 601 females. Among the females, 315 were reproductively active, carrying eggs or embryos in their marsupia (Supplementary Table S2). The body length [mm] was measured from the tip of the rostrum to the tip of the telson, excluding the spines. The body length was used later to counteract against all size specific parameters. *G. roeselii* has three to four dorsal spines that are important in defence against predators^36^. The number of spines as well as the area of these spines was measured. For this purpose, we measured the two outer sides (a and c) and their angles (γ) for each spine (see Supplementary Figure S1A). The area was then calculated using the formula$\:(a\: \times \:c\: \times \:\text{s}\text{i}\text{n}(\gamma\:\left)\right)/2$. As some of the specimens only had three spines the mean values for the spine area is used (see number of spines per site in Supplementary Figure S2).

The gills of amphipods are important for physiological homeostasis of oxygen and CO_2_ concentrations^37^, excretion of nitrogenous by-products, osmoregulation as well as mediating the intake of metals and contaminants^30,38,39^. The gill surface area [mm²] was measured on all six pairs of gills, using only one gill of a pair on the same side of the body^30^. Only in the case of a damaged gill, we used the respective gill from the opposite side of the body.

The first and second antennae in amphipods are used for finding food, habitat as well as communicating and interacting with potential partners for reproduction^40–43^. In gammarids, a female-produced pheromone (probably ecdysone or a related compound) deposited on the cuticle elicits male courtship behaviour through chemoreceptors on their second antennae^44^. The length of the antennae is often related to the population density and the sex ratio^30,45^. The first antennae were measured throughout the whole length whereas the second antenna was measured from the second segment to the tip as the first segment was difficult to dissect.

#### Male specific traits

Male gammarids exhibit pre-copulatory behaviour, known as amplexus, where the male grasps and guards a female using his gnathopods to secure the female before and during copulation^46,47^. This ensures that the male is in position to fertilize the female’s eggs immediately after she moults. We measured the propodus area of the first and second gnathopod as a proxy for clasping force of male gammarids, as it influences the male’s ability to securely hold females and outcompete rivals during pre-copulatory guarding. To do this, we carefully removed gnathopods from one side of the body and measured the propodus area (see Supplementary Figure S1B).

#### Female specific traits

In female crustaceans, both fecundity (i.e., the number of eggs) and egg size are critical reproductive traits and can be influenced by different factors such as pollution [e.g. *Asellus aquatics*^48^, *Echinogammarus marinus*^49^]. In *G. roeselii*, especially the size of the stream, conductivity and altitude as well as the thermal pollution influenced the number of eggs carried by females^30^. We therefore counted the number of eggs within the marsupium. The eggs were then carefully removed from the marsupium to evaluate the stage of each egg according to Jourdan et al.^30^. If different eggs showed signs of different stages the most common stage in this brood was taken. The longest (l) and the shortest (b) side of each egg at the broadest spot was measured and the volume was calculated using the formula for an ellipsoid: $\:\pi\:\times\:l\times\:{s}^{2}/6$ see ^32^.

#### Body length correction of traits

Phenotypic characters are often correlated with body size. We checked all traits for correlations with body length and corrected for body size accordingly. To do this, we first checked all traits for normal distribution with the ‘shapiro.test’ function and no parameter was normally distributed. Accordingly, we applied generalized additive models (GAMs) to each parameter using body length as covariate. GAMs allow for nonlinear relationships by applying smooth functions to covariates, capturing underlying patterns in the data. The degree of smoothness is controlled by the smoothing function, where a lower value enforces a more linear fit, while higher values allow for more flexibility. To fit the GAM model we used the ‘gam’ function in the package “mgcv”. We used the Restricted Maximum Likelihood (“REML”) method for smoothing parameter estimation. Model adequacy was assessed with the ‘gam.check’ function, which evaluates residual patterns and the adequacy of smoothness selection. The model was then used to predict the life-history parameters for the corresponding body length. For ‘egg volume’, we also included ‘embryonic developmental stage’ as an additional categorical factor to account for potential variation due to developmental differences, applying a similar correction for its effects. The residuals were then used in the further analysis. The mean measurements per MOTU are provided in Supplementary Table S3.

### Molecular methods

DNA extraction was carried out on 20 individuals per sampling site using the protocol of Montero-Pau et al.^50^. Two to three pereiopods were dissected from the left side of each individual and placed individually in 96 well-plates. 30 µL Lysis buffer made from 25 mM NaOH, 0.2 mM Na_2_EDTA and ddH_2_O was added to the samples. After sealing the plate it was incubated for 30 min at 95 °C and subsequently cooled to 4 °C for 5 min. A neutralizing buffer was added made up of 40 mM Tris HCL and ddH_2_O.

Using a polymerase chain reaction (PCR) the COI fragments were amplified. Different primer pairs were used as done by Grabowski et al.^28^ and Kabus et al.^34^. The corresponding primers to each individual/site are listed in Supplementary Table S1 in Kabus et al. ^34^. We used the universal primers LCO1490 and HCO2198^51^, UCOIF and UCOIR^52^ and COIGrF and COIGrR2^28^. PCR was carried out in a volume of 30 µL in 96 well-plates. The mastermix consisted of of 3.6 µL MgCl_2_ (25 mM), 3.0 µL 10 x Taq-Buffer (BioLabs), 0.4 µl BSA, 2.4 µL dNTPs (40mM), 0.4 µL Taq polymerase (Biolabs), each primer with a volume of 1 µL (10 mM), 14.2 µL of ddH_2_O and 4 µL DNA template. For the primer pair LCO1490/HCO2198 the protocol of Weiss et al.^53^ was applied: Initial denaturation at 94 °C (2 min), 36 cycles of denaturation at 94 °C (20 s), annealing at 46 °C (30 s) and elongation at 65 °C (1 min) and a final extension at 65 °C (5 min). For the primer pairs UCOIF/UCOIR and COIGrF/COIGrR2 the protocol of Mamos et al.^54^ was carried out: Initial denaturation at 94 °C (3 min), 35 cycles of denaturation at 94 °C (20 s), annealing at 50 °C (45 s) and elongation at 65 °C (1 min) and a final extension at 65 °C (2 min). PCR products were checked for successful amplification by running a 5 µl aliquot on a 1.0% agarose gel stained with GelRed^®^ (VWR). The amplified products were then sent to the Senckenberg Biodiversity and Climate Research Centre (SBiK-F) for sequencing. Geneious Prime software was then used to authenticate the sequences with a BLASTn search. Following this, the sequences were aligned using ClustalW and trimmed to a length of 522 bp. To correctly delimit the sequences to the known MOTUs, a neighbour-joining tree was calculated and sequences of cryptic *G. roeselii* already published by Grabowski et al.^28^ and Kabus et al.^34^ were used as a measure. Sequences acquired within this study are published online in the Barcode of Life System (BOLD) using their assigned IDs (see Supplementary Table S1).

### Characterizing environmental gradients

A range of environmental parameters were recorded to characterize the environmental conditions of each sampling site. These measurements were taken in situ and included: flow velocity [m s^− 1^] (Dostmann electronic P670), pH (Hach HQ40d multi, PHC201), conductivity [mg L^− 1^] (Hach HQ40d multi, CDC401), and oxygen concentration [mg L^− 1^] (Hach HQ40d multi, LDO101). Water samples from each sampling site were analysed for nitrate (NO_3−_), nitrite (NO_2−_) and phosphate (PO_4_^3−^) content using a photometer (FinwellPro for ponds; MDE GmbH & Co. KG, Germany). We additionally measured carbonate hardness (HCO_3_^−^) using a colorimetric kit (Merck MColortests).

To further assess anthropogenic impact on the stream and river characteristics, additional parameters of the dataset of Domisch, Amatulli^55^ which provides a freshwater-specific set of environmental variables across a 1 km river network grid. Domisch et al.^55^ provided information on land cover, climate, river topography and geological conditions from 1970 to 2000 using upstream accumulation techniques to obtain watershed contributions for each pixel. We considered the following variables: cultivated and managed vegetation (lc_avg_07; “*cultivated landcover*”), urban/built-up landcover (lc_avg_09; “*urban landcover*”), annual mean temperature (Bioclim1; “*annu. mean temperature*”), annual precipitation (Bioclim12; “*annu. precipitation*”) and flow length (size of the catchment upstream of the sampling site as the sum of 1 km contributing grid cells). The mean of these variables was weighted over the distance of the upstream catchment up to the sampling site for a 1 km grid cell of a terrestrial dataset (see Supplementary Table S4).

We furthermore took sediment samples at each sampling site to assess the long-term pollution load using in vitro assays. We used the bioassays to assess specific toxicological endpoints:: Yeast Estrogen Screen for estrogenic [YES^56^], Yeast Dioxin Screen for dioxin-like activity [YDS^57,58^] and a Microtox assay with *Aliivibrio fischeri* for assessing baseline toxicity [Microtox^59,60^]. The detailed description of the in vitro assays is given in Kabus et al.^34^. See Supplementary Table S5 for raw data of the effect-based measurements.

### Statistical analysis

Based on all the recorded environmental variables (see Supplementary Table S4-S5), we determined the environmental gradients by applying a principal component analysis (PCA). The PCA was conducted with the function ‘dudi.pca’ within the R package “ade4” [Ver. 1.7–19^61^]. The parameters were standardized and centred (z-score transformed). We conducted a varimax rotation to increase the interpretation of the PCA and maximize the sum of the variance of the squared loadings. Rotated PC1 (rPC1) explains 25.51 % of the variation within the dataset, whereas rPC2 explains 15.02 % (see Supplementary Figure S2 and Table S6). rPC1 is primarily associated with high negative loadings of conductivity (-0.787), nitrite (-0.605), carbonate hardness (-0.796) and mean annual temperature (-0.683), whereas it has positive loadings with flow length (0.663) and annual precipitation (0.664). PC2 is negatively loaded with altitude (-0.740) and pH (-0.687). The full list of axis loadings (rPC1 - rPC4) is attached in the Supplementary Table S6. We used the function ‘PCAtest’ in the package “PCAtest” (Ver. 0.0.1) to check for a significant contribution of the rPCs^62^. Only rPC1 and rPC2 showed significant contributions (*p* < 0.05) to explaining variance in the environmental data, and thus were retained for further analysis.

In order to investigate the potential differences in various phenotypic traits among different populations and between MOTUs, we employed linear modelling techniques using the ’lm’ function in R. Separate linear models were constructed for each phenotypic trait to isolate the effects specific to each variable. In these models, we included rPC1 and rPC2 as continuous explanatory variables. Additionally, MOTU and sex were included as categorical predictors. Interactions between rPC1, rPC2, and the categorical predictors were also considered in the models. To evaluate the statistical significance of explanatory variables, we conducted Type II ANCOVAs using the Anova function from the “car” package^63^. Predicted effects were calculated using the ‘ggpredict’ function in the “ggeffects” package^64^, which allows for the estimation of average trait values while accounting for covariate effects. We verified model assumptions by visually inspecting diagnostic plots, including Q-Q plots for normality of residuals and residuals vs. fitted values to assess homoscedasticity. These steps ensured that our models met the necessary assumptions for accurate interpretation of the results.

To further evaluate the variance within and between MOTUs we applied a Discriminant Analysis of Principal Components (DAPC) on body-size corrected phenotypic traits for both male and female individuals using the R package “adegenet” (Ver. 2.1.10.). DAPC is a multivariate method particularly suited for identifying and describing clusters of phenotypically similar individuals by combining the advantages of PCA and discriminant analysis. The function ‘dapc’ performs a discriminant analysis on the basis of a PCA. MOTU was set as the grouping factor, and separate analyses were conducted for males and females to assess potential sex-based differences. The discriminant analysis maximizes the between-group/MOTU variation while minimizing within-group variation to best separate the groups/MOTUs. Visualization of the DAPC results through scatterplots of discriminant functions and loading plots allowed us to identify the traits contributing most significantly to the potential divergence among MOTUs. These results complement our linear modelling approach, providing a robust framework to evaluate and interpret the structure of phenotypic variation within and among MOTUs. All statistical analyses and data visualizations were performed using R version 4.1.0^65^.

## Results

### MOTU specific phenotypes

**Fig. 3:**
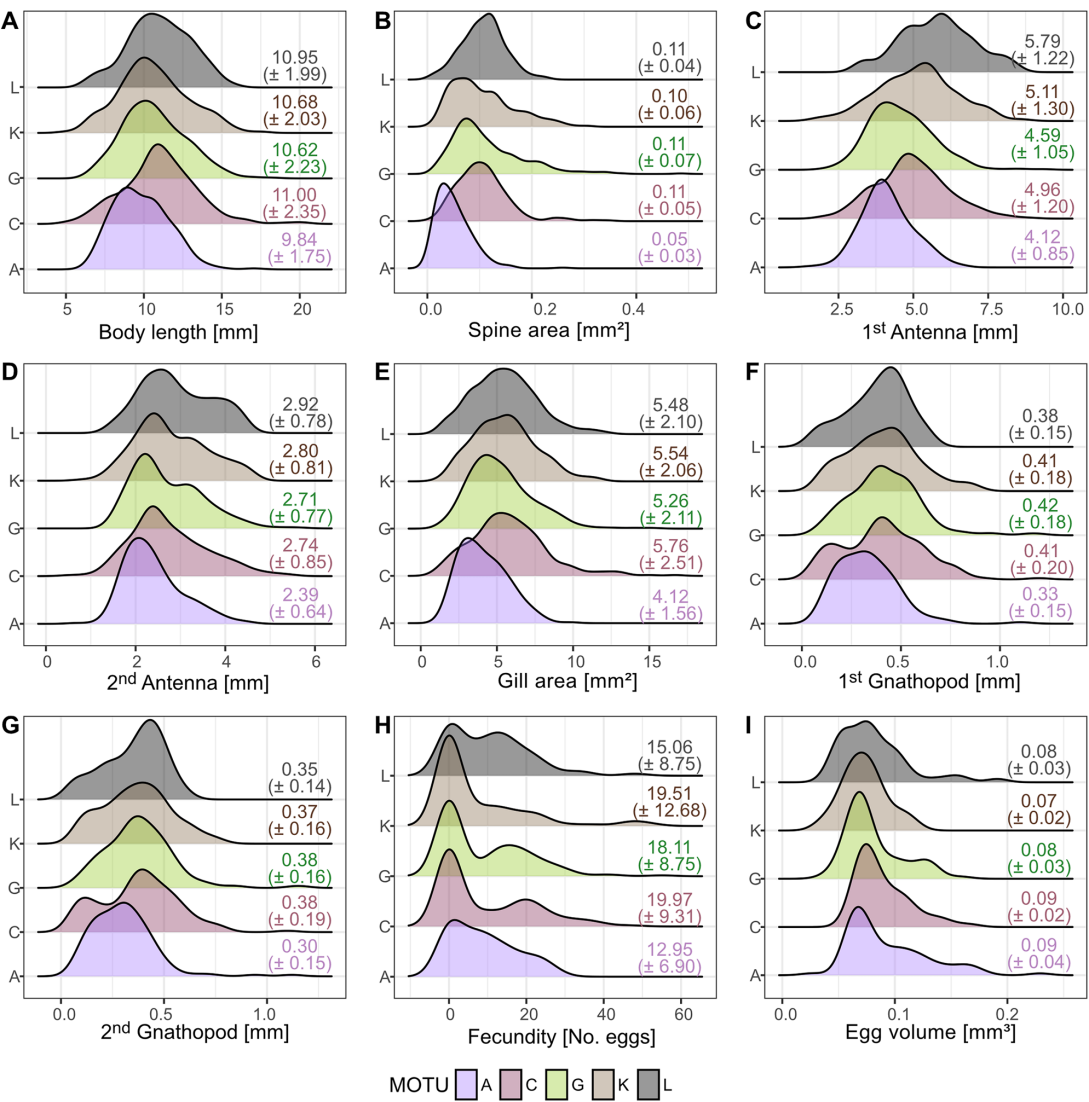
Ranges of quantified morphological and life-history traits for five MOTUs of the *G. roeselii* species complex. The histograms represent the raw data of the measured traits. The mean values ± standard deviations are displayed next to each MOTU.

The descriptive quantification of phenotypic traits shows that all investigated MOTUs in the *G. roeselii* species complex overlap phenotypically, supporting that these species are and cannot be distinguished from each other on the basis of the measured traits (Fig. 3, see also Supplementary Table S3). The results indicate only marginal shifts in morphological traits, with MOTU A exhibiting a tendency towards smaller overall body size compared to the other MOTUs. This trend is similarly reflected in the spine area and the length of the 1st antennae.

### Phenotypic differentiation across gradients

**Fig. 4:**
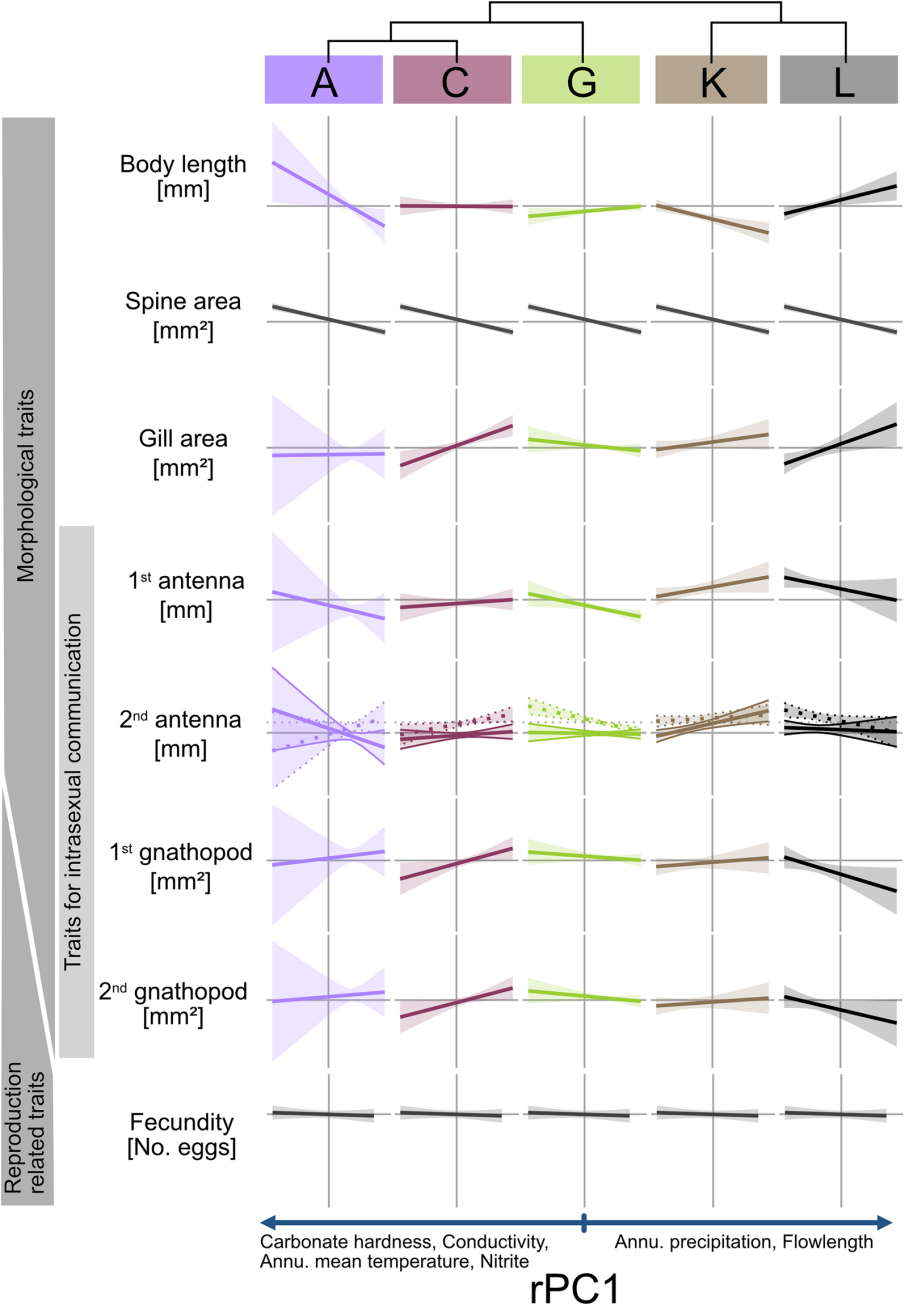
Linear trends of all measured traits to rPC1 for each MOTU. The linear trend of all morphological and reproductive related traits in divided in MOTU identity, represented by the colours, is displayed. The horizontal line represents the mean value of the trait for all MOTUs combined. If only an environmental effect was found, a repeated graph of the overall effect is depicted in dark grey (see spine area and fecundity). If sex and MOTU were significant in the ANOVA, the trend is split in male (dotted lines) and female (full lines). The shaded area represents the 95% confidence interval. A positive trend is correlated with a positive rPC1 (high flow length and annual precipitation and low carbonate hardness, conductivity, annual mean temperature and nitrite content). At the top a simplified phylogenetic tree depicts the genetic connection of the MOTUs.

**Fig. 5:**
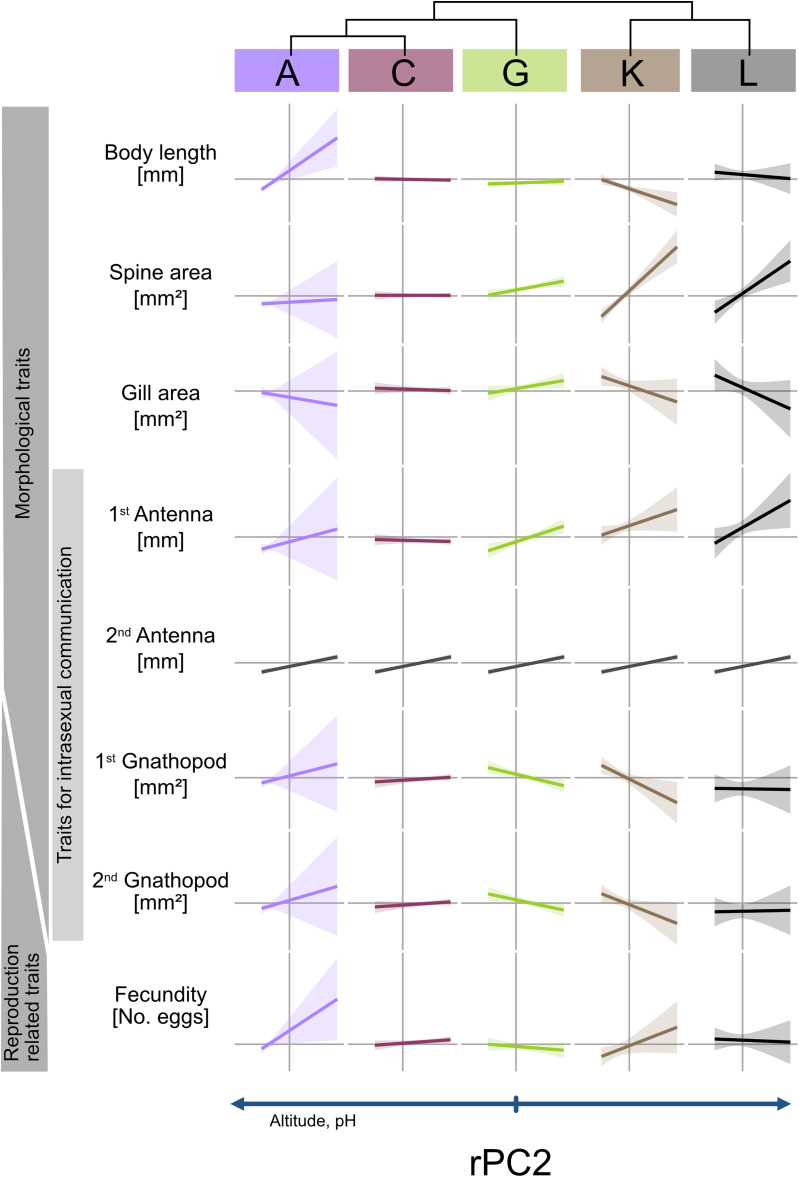
Linear trends of all measured traits to rPC2 for each MOTU. The linear trend of all morphological and reproductive related traits in divided in MOTU identity, represented by the colours, are displayed. The horizontal line represents the mean value of the trait for all MOTUs combined. The shaded area represents the 95% confidence interval. A positive trend is correlated with a positive rPC2 (low altitude and pH). If only an environmental effect was found, a repeated graph of the overall effect is depicted in dark grey (see 2nd antenna). At the top a simplified phylogenetic tree depicts the genetic connection of the MOTUs.

Our linear models predominantly revealed unique phenotypic differentiation of MOTUs along the environmental gradients (indicated by significant interaction terms MOTU × rPC; see Table 1; Figs. 4 and 5). Only spine area and fecundity along rPC1 and the 2nd antenna along rPC2 showed convergent phenotypic differentiation (indicated by non-significant interaction terms, but significant impact of rPC1/rPC2). For the second antenna, we further found a sex and MOTU specific differentiation along rPC1.

We observed some unique patterns of differentiation across rPC1, for example, in body length and gill area. Body length increased with higher mean temperatures (indicated by negative rPC1) in MOTU A and K but decreased in MOTU G and L (Fig. 4). Similarly, gill area increased in MOTU C, K, and L with increasing rPC1, but declined in MOTU G. A convergent pattern of phenotypic changes along rPC1 was found for the spine area. The spine area increased in all MOTUS with increasing rPC1, i.e. in warm areas with high conductivity and nitrite we observed large spines.

**Table 1:**
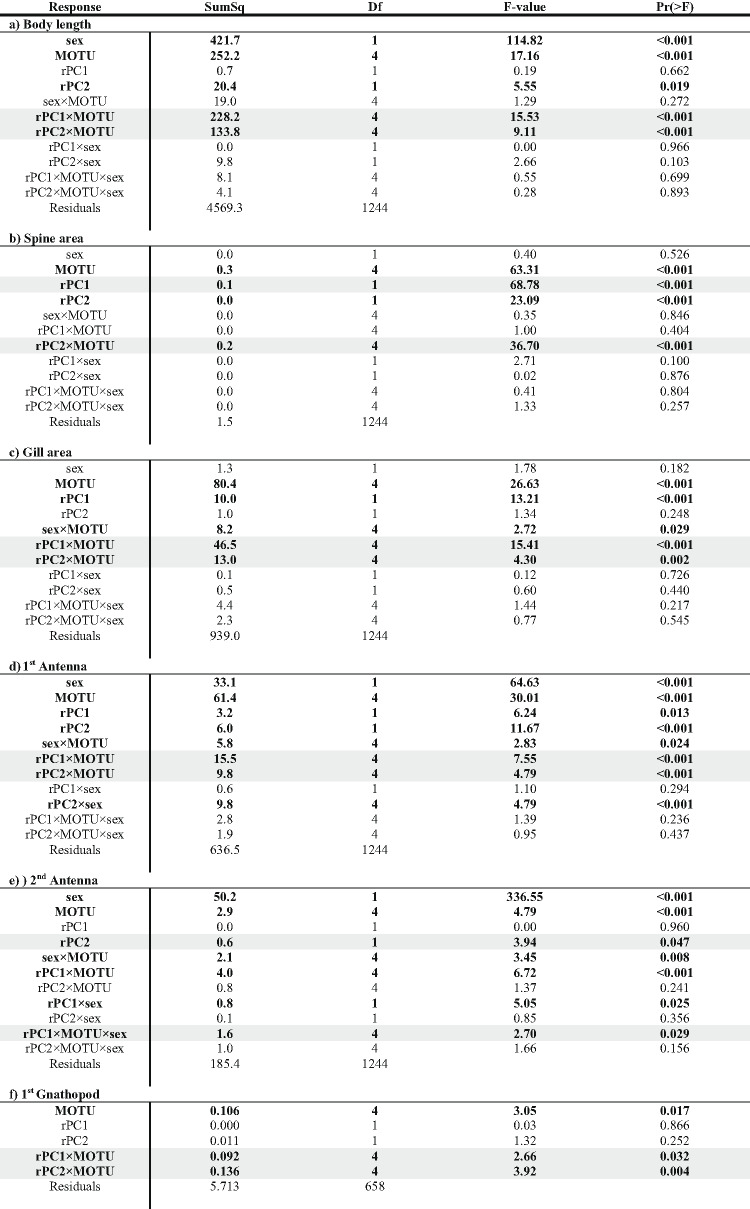

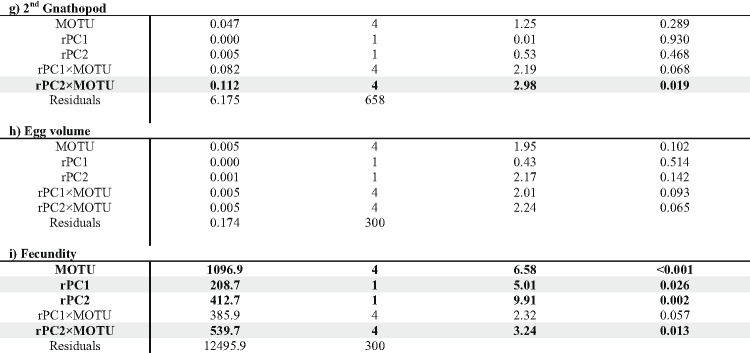
ANOVA results on nine body length corrected phenotypic traits: significant results (< 0.05) that are also graphically shown in Figs. 4 and 5 are highlighted in grey. Other significant results are displayed in bold letters and numbers. Analyzed traits include (a) body length, (b) spine area, (c) gill surface area, (d) 1st antenna, (e) 2nd antenna in both sexes; (f) 1st gnathopod; (g) 2nd gnathopod in males; and (h) egg volume and (i) fecundity in females.

A look at the second environmental gradient (rPC2) also revealed predominantly unique patterns of differentiation. For example, there were clear increases in the spine area in the southern MOTUs K and L from high altitude sites to low altitude sites; both MOTUs also showed comparable reduction in gill area and body length at low altitude sites. These differentiations were less pronounced in the more northern MOTUs A, C and G.

### MOTU-specific sexual dimorphism

**Fig. 6:**
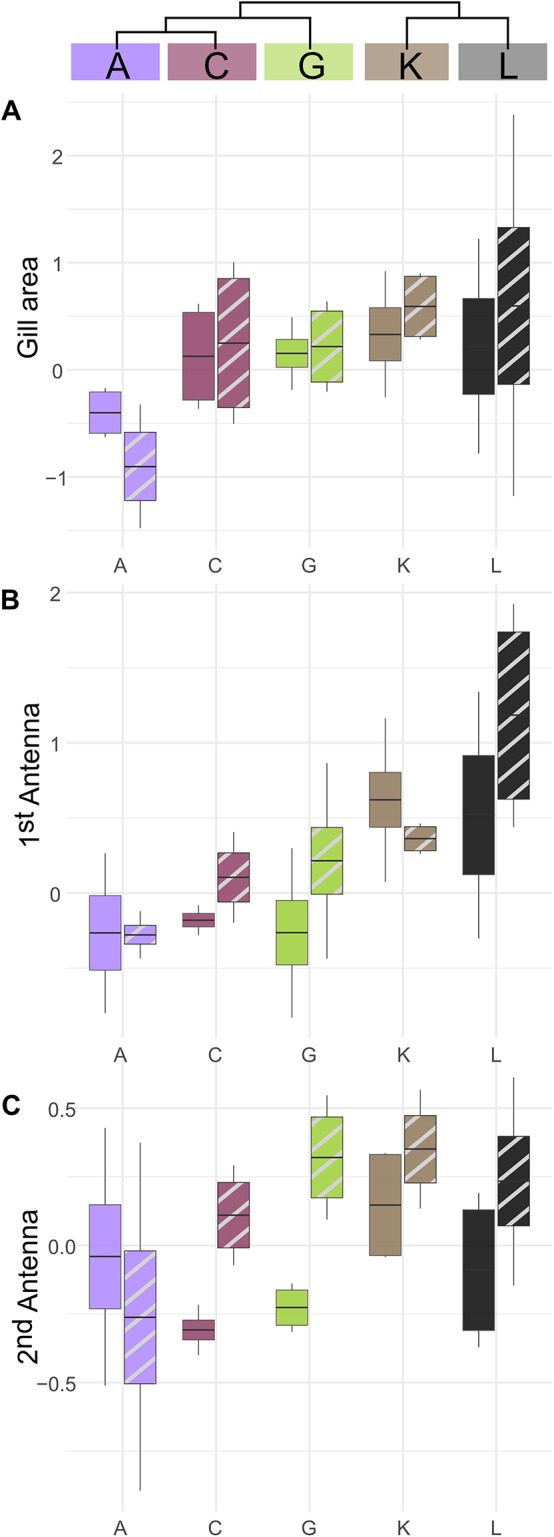
Sexual dimorphism of selected phenotypic traits within each MOTU The y-axis shows body-size–adjusted residuals. Whiskers indicate the 95% confidence interval. Colours denote different MOTUs; solid boxes represent females and striped boxes represent males.

For some traits, we also found a MOTU-specific sexual dimorphism (indicated by a significant sex × MOTU term). This was the case, for the gill area (Fig. 6A), 1st antenna (Fig. 6B) and 2nd antenna (Fig. 6C). Most of the measured traits were larger in males than in females despite body size correction. However, for the gill area, the males of MOTU A have a smaller gill area than the females (Fig. 6A). A feature with strong sexual dimorphism are the 1st and 2nd antennae, which are usually larger in males (Fig. 6B-C). However, we also found MOTU-specific differences here. For example, sexual dimorphism in the antennae was hardly present in MOTU A. In MOTU K, the 1st antenna was smaller in males than in females.

### Within and between MOTU variation

**Fig. 7:**
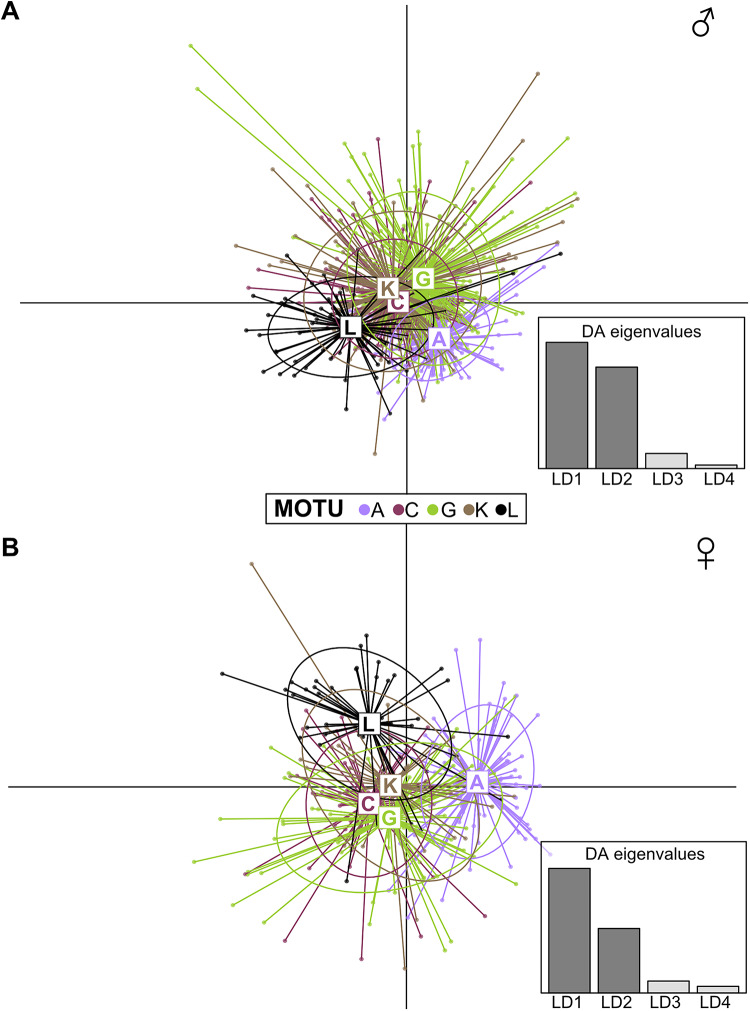
Discriminant analysis of five different *G. roeselii* MOTUs based on (**A**) male and (**B**) female phenotypic traits. Each MOTU is represented by a unique colour, with circles indicating the 95% confidence interval of each MOTU. For females, we only considered egg-carrying females.

The DAPC analysis considered the total phenotypic variance and confirmed the large overlap in traits between the MOTUs. Specifically, we retained the first two principal components in the DAPC analysis, capturing 58.2% (males) and 44.6% (females) of the total variance (see Supplementary Table S7 for loadings of PCA used in discriminant analysis). Among the discriminant functions, the first and second axes explained 51.3% and 41.2% (Fig. 7A, Supplementary Table S8) in males and 60.0% and 31.1% in females (Fig. 7B, Supplementary Table S7). Group assignment accuracy was low, with an average classification success rate of 49.9% in females and 56.5% in males across MOTUs (see Supplementary Table S9), confirmed through cross-validation, suggesting high within-MOTU variation. Visualization of the DAPC scatterplot further showed no separation between MOTUs, with all 95% inverval circles of MOTUs clearly overlapping. The traits with the strongest loadings on the primary discriminating functions were the propodus area of the first gnathopod (males) and spine area (females), indicating that these phenotypic traits differed most between MOTUs (for all loadings of traits see Supplementary Table S10).

## Discussion

The first in-depth phenotypic characterization of the cryptic species complex *G. roeselii* revealed that, despite 18 million years of diversfication, the cryptic species status was consistently preserved. In agreement with our hypothesis, morphological crypsis evolved due to differntial selection and phenotypic differentiation across environmental gradients although it was rarely convergent and predominantly specific to MOTUs. Revisiting our theoretical framework for phenotypic differentiation (Fig. 1), the majority of responses aligned with the adaptive trajectory outlined in Fig. 1F, while a subset mirrored convergent pattern of trait differentiation (Fig. 1E), maintaining cryptic morphological stasis in both cases.

### Phenotypic differentiation along environmental gradients

We expected to find convergent differentiations of MOTUs along the environmental gradients, which was hardly confirmed. In theory, similar selective pressures in different environments (e.g., temperature, resource availability) could lead to similar adaptive solutions, even in genetically distinct species/lineages^2^. This is particularly expected in a cryptic species complex, where long-term exposure to similar selective forces obviously favored very similar adaptive responses over millions of years, which has prevented phenotypic differentiation^3^. However, only a few traits, such as the spine area – a trait that serves as a predator defense^36^ - showed convergent changes across all MOTUs. The convergent change in spine length likely results from strong predation pressure selecting for optimal defense strategies along the environmental gradient. In contrast, other traits may be more influenced by MOTU-specific ecological interactions, developmental constraints, or evolutionary histories, leading to unique adaptive responses. For example, even traits, such as body length, which would be expected to follow globally observed patterns (e.g., the temperature-size rule, with body size decreasing as annual mean temperature rises;^66,67^), did not exhibit this pattern in some cases (e.g., MOTUs A and K), while it does in others. Reasons for the lack of convergent response could be the ecological or physiological constraints, such as MOTU-specific metabolic rates, local temperature regimes or habitat conditions^68,69^. Another explanation for the absence of convergent responses is that MOTUs were not exposed to truly identical environmental conditions. The environmental gradients identified are based on absolute values, meaning that both locally specific combinations of selection pressures (see below) and absolute values themselves may not have been sufficient to detect convergent evolution. For instance, while certain trends were observed at higher temperatures (e.g., MOTU K and L) and lower temperatures (e.g., MOTU C), these represent only a subset of broader patterns seen at a larger scale. Without assessing all MOTUs across the full temperature range, the influence of this environmental factor may remain obscured. Addressing this uncertainty would require either ecologically risky transplantation experiments (which is hardly acceptable due to the risk of faunal mixing) or multigenerational laboratory studies. Such experiments would furthermore exclude the influence of other interacting selective pressures, like predation, resource availability or inter-specific competition within a aquatic community, which could favor either smaller or larger body sizes at higher temperatures^7,70^. For example, the availability of food can have a direct effect body size of amphipods which can explain some of the lack in convergent phenotypic responses here^7,71^. Gill surface area emerged as a consistently responsive trait, exhibiting a pronounced reduction in three of the five MOTUs along rising conductivity gradients – a pattern aligning with prior empirical evidence linking diminished gill area to osmoregulatory optimization under ionic stress^30,32^. This repeatedly observed response underscores the role of adjusting gill morphology as a way to cope with stressful conditions in chemically contaminated or high-salt waters^72^. Such functional plasticity makes gill modifications a useful bioindicator for tracking changes in freshwater ecosystems exposed to anthropogenic pressures. Body size-corrected gnathopod size inversely scales with body size along the environmental PC1, suggesting proportional gnathopod reduction accompanies body size increases. This trade-off in resource allocation suggests environments favoring larger body size (e.g., due to resource availability, temperature, or predation) reduce the need for proportionally larger gnathopods, as their absolute size remains sufficient for mate guarding^45,73^.

Our framework for characterizing environmental gradients, while a commonly used tool [e.g.,^74–76^], is inherently limited in its ability to account for the full complexity of ecological variability present in the system. Such fine-scale environmental variability (e.g. predation, resource and microhabitat availability) can drive localized adaptative responses^77–79^, leading to divergent phenotypic responses even when overall environmental gradients (e.g., temperature or river size) appear similar at a broader scale. Consequently, the expected uniform convergence of traits under shared selective pressures may be obscured by the heterogenicity and changing nature of these aquatic habitats, however, it may result in overall phenotype similarity among species^80^.

A limitation of our study is that the geographical clustering of MOTUs results in partly non-overlapping environmental distributions, making it difficult to fully separate MOTU from environmental effects. Interaction patterns with environmental gradients should therefore be interpreted with caution, as nonlinear responses may generate apparent differences without true MOTU-specific effects. Because most MOTUs do not co-occur under identical conditions, this limitation is not easily resolved. Future studies focusing on MOTUs in overlapping habitats at smaller spatial scales will be essential to verify whether the observed patterns represent genuine MOTU × environment interactions.

### Sexual dimorphism and its MOTU-specific variation

We found that traits subject to strong sexual selection were differentially sexually dimorphic between MOTUs, suggesting altered selection regimes. For instance, while gammarids typically exhibit pronounced sexual dimorphism in the antennae [males > females;^30^, MOTU A lacked this pattern in both antennae and MOTU K in the first antenna, with males having smaller antennae overall. The reversed sexual dimorphism in MOTU A’s antennae (females > males) may reflect a female-biased sex-ratio reducing the male-male competition (e.g., mate monopolization). This pattern aligns with findings in the subterranean amphipod *Niphargus* where populations with female-biased sex-ratio exhibited reduced sexual dimorphism due to weakened male-male competition^81^. In contrast, in populations with balanced sex-ratio, males are under stronger sexual selection, leading to more pronounced dimorphism. Such a reduction or even reversal of sexual dimorphism challenges the assumption that sexual dimorphic traits and sexual roles are static, suggesting mating systems in amphipods may exhibit adaptive plasticity under shifting ecological or demographic regimes. An alternative explanation for the change of sexual dimorphism in the antennae could be that ecological constraints (e.g., high flow, predation) may favor a streamlined male morphology, reflecting a potential trade-off between sexual selection and hydrodynamic efficiency^81,82^. Future work could test whether this reversal indeed correlates with habitat-specific sex-ratios or hydrodynamic performance trade-offs.

However, if such patterns of sexual dimorphism are shown to be consistent within certain MOTUs (e.g., shorter 1st antennae in males than females in MOTU K), they could serve as phenotypic criteria for distinguishing MOTUs from one another.

### Higher within- than between-MOTU differentiation

We observed greater differentiation within than between MOTUs, which is consistent with the fact that most MOTUs are confined to relatively small geographic areas, often limited to a single river system (except for MOTU C). Geographical barriers, such as mountain ranges, are known drivers for allopatric differantiation^83^ and explain genetic differetiation within *G. roeselii* species complex. These barriers limit gene flow promoting genetic divergence through drift or divergent selection which can enhance cryptic speciation^26,84^. Within these systems, however, they can span diverse habitats – from headwaters to larger river reaches and estuaries or deltas. Variations in flow, temperature and nutrient input are likely to impose different selective pressures on local populations as shown for gammarids^30,85,86^. Meanwhile, selection pressures in neighboring river systems may be more similar, allowing for the maintenance of a range of phenotypes within a single MOTU. Thus, the interplay between local adaptation (driving within-MOTU differentiation) and broad-scale stabilizing selection (maintaining phenotypic consistency across similar environments) illustrates the complex dynamics shaping cryptic diversity in these aquatic systems.

An important point is that overlapping trait distributions among MOTUs are not necessarily unique to the *G. roeselii* cryptic species complex. Even morphologically well-distinguishable species within the same genus, such as *G. fossarum* and *G. pulex*, may show strong inter-population variability that overlaps with traits of *G. roeselii* (e.g., body size, brood size, or age at maturity). This indicates that phenotypic overlap is not in itself evidence of cryptic diversity but may reflect a more general feature of gammarid amphipods – and potentially other taxa. Our results therefore contribute to the broader discussion of trait variability across taxonomic levels and highlight the importance of integrating trait-based and molecular approaches in species delineation.

### Implications for future research and conservation

Conservation efforts are not evenly distributed across the tree of life. Vertebrates, particularly mammals and birds, tend to receive disproportionately more legal protection and conservation attention than invertebrates, plants, fungi, or other less charismatic groups^87^. Moreover, conservation frameworks prioritizing species-level taxonomy often fail considering cryptic diversity, exposing species that lack diagnostic morphological divergence to unmitigated threats^88^. Efforts to change this date back to the 1980s, when the idea of incorporating evolutionary significant units first emerged^89,90^. While these concepts were initially focused on charismatic zoo animals and their subspecies, they can also be applied to cryptic invertebrate species complexes. The MOTUs of the cryptic species complex *G. roeselii* are endemic to small geographic regions and our study shows unique adaptive responses to environmental variables undergoing rapid changes in times of global change. However, so far *G. roeselii* has proved to be slightly more resilient to various stressors compared to other aquatic invertebrates^91^. While research remains preliminary, current findings suggest that tolerance levels are similar across MOTUs of *G. roeselii*^32,35^. Combined with their prevalence in environmentally stressed habitats, this points to a certain capacity for adaptive response across the species complex.

A critical threat for the species complex might be human-driven admixture of MOTUs – disrupting historical geographic structuring – mirroring invasive species dynamics^92^ at a microevolutionary scale and eroding lineage-specific adaptations. To explore the consequences of overcoming geographical barriers, it is crucial to understand the extent to which MOTUs would interbreed. However, there is little research on this in cryptic amphipods so far. Some evidence for mate discrimination in cryptic amphipods comes from Lagrue et al.^12^ who found that while pre-copulatory pair formation was random among genetically similar individuals of *G. fossarum* and *G. pulex*, it was rare between highly divergent MOTUs under natural conditions (> 4%). The authors showed that amphipods from different MOTUs could mate successfully in captivity, producing viable eggs, even when genetic difference reached up to 16% in the COI gene. This suggests that cryptic diversity in amphipods may be driven more by pre-zygotic isolation through mate discrimination. The *G. roeselii* complex also has contact zones of different MOTUs, for example in south-eastern Albania. Interestingly, Grabowski et al.^28^ reported co-occurring MOTUs E and J (site 13 in their study, corresponding to site 11 here) and MOTUs E and G (site 11 vs. 19) during sampling in 2006–2008. However, our 2021 surveys detected only MOTU G at the same two sites. This shift suggests ongoing or recent replacement dynamics within the species complex, highlighting the regions value as a natural laboratory for studying cryptic speciation and ecological competition within a cryptic species complex.

An emerging field of research involves understanding divergent parasite infection patterns within cryptic species complexes – a dimension of ecological complexity rarely explored so far. However, some first research showed that cryptic diversity in native amphipods (*G. pulex* / *G. fossarum*) created uneven susceptibility to acanthocephalan parasite infections, influencing inter-MOTU competition and native population dynamics^93,94^. For acanthocephalans, their multi-host life cycles (often involving highly mobile birds or fish as final hosts) may enable these parasites to overcome geographical barriers more effectively than their intermediate gammarid hosts. Investigating how parasite communities assemble across such species complexes – particularly in ecosystems altered by chemical pollution – offers critical insights into how co-evolving host-parasite dynamics shape resilience, competition, and community structure under environmental stress^31,93,95,96^.

## Conclusion

Our study highlights the interplay between environmental gradients, local adaptation, and sexual selection within a cryptic species complex. The cryptic species complex of *G. roeselii* proved to be an insightful model for examining the interplay between adaptive divergence and biogeographic history in shaping current biodiversity patterns. Our study revealed that, despite phenotypic differentiation along environmental gradients, the *G. roeselii* species complex remains consistently cryptic, probably shaped by ecological interactions and evolutionary histories. Additionally the partially reversed sexually dimorphic characters in MOTU A leads to the assumption that sexually selected traits are not as static but rather exhibit dynamic variation influenced by changing ecological or demographic factors.

## Supplementary Information

Below is the link to the electronic supplementary material.

Supplementary Material 1

Supplementary Material 2

## Data Availability

All phenotypic data generated or analysed during this study are included in this published article (and its Supplementary Information files) and the genetic sequences are available at BOLD Systems accessible via https://v4.boldsystems.org/ through the accession numbers GROLH001-24 – GROLH700-24.
