# Supplementary material for: Morphological crypsis within a crustacean species complex is driven by within-species phenotypic diversification
Source: Sci Rep. 2025 Dec 2;15:43020. doi: 10.1038/s41598-025-29270-1 (PMC12675779; doi:10.1038/s41598-025-29270-1)
Supplement: Supplementary file 2 — Supplementary Material 2 [file 41598_2025_29270_MOESM2_ESM.docx]

**Supplementary Material: “Morphological crypsis within a crustacean species complex is driven by within-species phenotypic diversification”**

**Table S1 Sampling sites and locations visited as well as the BOLD IDs for each site.** DE = Germany, AL = Albania, GR = Greece, SL = Slovenia

| **Site** | **MOTU** | **Country** | **River basin** | **Location North [decimal °]** | **Location East [decimal °]** | **BOLD ID** |
| --- | --- | --- | --- | --- | --- | --- |
| 1 | C | DE | Kinzig | 50.390583 | 9.400805 | GROLH001-24 - GROLH020-24 |
| 2 | C | DE | Kinzg | 50.346335 | 9.552046 | GROLH021-24 - GROLH040-24 |
| 3 | C | SL | Danube | 46.6093666 | 14.9735864 | GROLH041-24 - GROLH060-24 |
| 4 | C | SL | Danube | 46.5443485 | 15.5047118 | GROLH061-24 - GROLH080-24 |
| 5 | C | SL | Danube | 46.5896778 | 16.1778965 | GROLH081-24 - GROLH100-24 |
| 6 | C | SL | Danube | 46.522732 | 16.278691 | GROLH101-24 - GROLH120-24 |
| 7 | C | SL | Danube | 46.522467 | 16.150638 | GROLH111-24 - GROLH140-24 |
| 9 | C | SL | Danube | 46.379587 | 15.939002 | GROLH141-24 - GROLH160-24 |
| 10 | G | AL | Drin | 42.024833 | 19.519133 | GROLH161-24 - GROLH180-24 |
| 11 | G | AL | Shkumbin | 41.0710747 | 20.466527 | GROLH181-24 - GROLH200-24 |
| 12 | G | AL | Shkumbin | 41.0640253 | 20.5659157 | GROLH201-24 - GROLH220-24 |
| 13 | G | AL | Drin | 40.9007099 | 20.6871298 | GROLH011-24 - GROLH240-24 |
| 15 | G | AL | Drin | 41.067533 | 20.64423 | GROLH241-24 - GROLH260-24 |
| 16 | G | AL | Devolli | 40.797563 | 20.718132 | GROLH261-24 - GROLH280-24 |
| 18 | G | AL | Devolli | 40.5243611 | 20.7027654 | GROLH281-24 - GROLH300-24 |
| 19 | G | AL | Devolli | 40.7078978 | 20.8713875 | GROLH 101-24 - GROLH320-24 |
| 20 | G | AL | Prespa | 40.8614197 | 20.940791 | GROLH311-24 - GROLH340-24 |
| 21 | G | GR | Prespa | 40.745446 | 21.114912 | GROLH341-24 - GROLH360-24 |
| 23 | A | GR | Papadia Lake | 40.831339 | 21.541287 | GROLH361-24 - GROLH380-24 |
| 24 | A | GR | Papadia lake | 40.840163 | 21.584415 | GROLH381-24 - GROLH400-24 |
| 25 | A | GR | Vegoritida lake | 40.728311 | 21.681089 | GROLH401-24 - GROLH420-24 |
| 26 | A | GR | Vegoritida lake | 40.738977 | 21.756498 | GROLH411-24 - GROLH440-24 |
| 27 | A | GR | Kastoria lake | 40.515293 | 21.255458 | GROLH441-24 - GROLH460-24 |
| 28 | A | GR | Kastoria lake | 40.435215 | 21.270994 | GROLH461-24 - GROLH480-24 |
| 29 | A | GR | Kastoria lake | 40.332211 | 21.411812 | GROLH481-24 - GROLH500-24 |
| 30 | L | GR | Pinios | 39.530766 | 21.702878 | GROLH501-24 - GROLH520-24 |
| 31 | L | GR | Pinios | 39.542354 | 21.613207 | GROLH511-24 - GROLH540-24 |
| 33 | L | GR | Pinios | 39.533338 | 21.925948 | GROLH541-24 - GROLH560-24 |
| 34 | K | GR | Rentiniotoikos Onochonos | 39.1970624 | 22.0328946 | GROLH561-24 - GROLH580-24 |
| 35 | K | GR | Sofaditis | 39.120757 | 22.158304 | GROLH581-24 - GROLH600-24 |
| 37 | K | GR | Spercheios | 38.93871 | 22.162333 | GROLH601-24 - GROLH620-24 |
| 38 | K | GR | Spercheios | 38.897608 | 22.317396 | GROLH611-24 - GROLH640-24 |
| 40 | K | GR | Spercheios | 38.813142 | 22.49172 | GROLH641-24 - GROLH660-24 |
| 41 | K | GR | Sofaditis | 39.094976 | 22.194814 | GROLH661-24 - GROLH680-24 |
| 42 | L | GR | Pinios | 39.878257 | 22.584298 | GROLH681-24 - GROLH700-24 |

**Table S2 Number of individuals used in the statistical analysis.**

| **Site** | **MOTU** | **Number of Individuals** | **Males**  **[No.]** | **Females [No.]** | **Eggcarrying females [No.]** | **Eggcarrying females [%]** | **Max. No. of spines** | **Min. No. of spines** |
| --- | --- | --- | --- | --- | --- | --- | --- | --- |
| **1** | C | 37 | 20 | 17 | 10 | 58.82 | 4 | 3 |
| **2** | C | 34 | 15 | 19 | 12 | 63.16 | 4 | 3 |
| **3** | C | 39 | 19 | 20 | 6 | 30.00 | 4 | 3 |
| **4** | C | 26 | 18 | 8 | 7 | 87.50 | 4 | 3 |
| **5** | C | 36 | 20 | 16 | 11 | 68.75 | 4 | 3 |
| **6** | C | 38 | 18 | 20 | 5 | 25.00 | 4 | 3 |
| **7** | C | 40 | 20 | 20 | 8 | 40.00 | 4 | 3 |
| **9** | C | 40 | 20 | 20 | 6 | 30.00 | 4 | 3 |
| **10** | G | 37 | 20 | 17 | 6 | 35.29 | 4 | 2 |
| **11** | G | 38 | 20 | 18 | 16 | 88.89 | 3 | 3 |
| **12** | G | 38 | 20 | 18 | 9 | 50.00 | 3 | 3 |
| **13** | G | 39 | 20 | 19 | 0 | 0.00 | 4 | 3 |
| **15** | G | 37 | 19 | 18 | 12 | 66.67 | 4 | 3 |
| **16** | G | 30 | 20 | 10 | 8 | 80.00 | 3 | 2 |
| **18** | G | 38 | 19 | 19 | 8 | 42.11 | 3 | 3 |
| **19** | G | 30 | 20 | 10 | 9 | 90.00 | 3 | 3 |
| **20** | G | 38 | 20 | 18 | 0 | 0.00 | 3 | 3 |
| **21** | G | 38 | 20 | 18 | 12 | 66.67 | 3 | 3 |
| **23** | A | 38 | 19 | 19 | 16 | 84.21 | 3 | 2 |
| **24** | A | 36 | 20 | 16 | 12 | 75.00 | 4 | 2 |
| **25** | A | 35 | 18 | 17 | 12 | 70.59 | 3 | 3 |
| **26** | A | 38 | 19 | 19 | 6 | 31.58 | 4 | 3 |
| **27** | A | 38 | 20 | 18 | 10 | 55.56 | 4 | 2 |
| **28** | A | 40 | 20 | 20 | 15 | 75.00 | 4 | 3 |
| **29** | A | 39 | 20 | 19 | 16 | 84.21 | 3 | 3 |
| **30** | L | 39 | 19 | 20 | 13 | 65.00 | 4 | 3 |
| **31** | L | 32 | 19 | 13 | 9 | 69.23 | 3 | 2 |
| **33** | L | 38 | 20 | 18 | 9 | 50.00 | 3 | 3 |
| **34** | K | 36 | 20 | 16 | 11 | 68.75 | 3 | 3 |
| **35** | K | 37 | 19 | 18 | 6 | 33.33 | 3 | 3 |
| **37** | K | 38 | 20 | 18 | 12 | 66.67 | 3 | 3 |
| **38** | K | 29 | 14 | 15 | 1 | 6.67 | 4 | 3 |
| **40** | K | 32 | 20 | 12 | 2 | 16.67 | 3 | 3 |
| **41** | K | 37 | 19 | 18 | 2 | 11.11 | 4 | 3 |
| **42** | L | 39 | 19 | 20 | 18 | 90.00 | 3 | 3 |
|  |  | | | | | |  |  |
|  | **Min** | 26 | 14 | 8 | 0 | 0 |  |  |
|  | **Max** | 40 | 20 | 20 | 18 | 90 |  |  |
|  | **All** | 1274 | 673 | 601 | 315 | 90 |  |  |


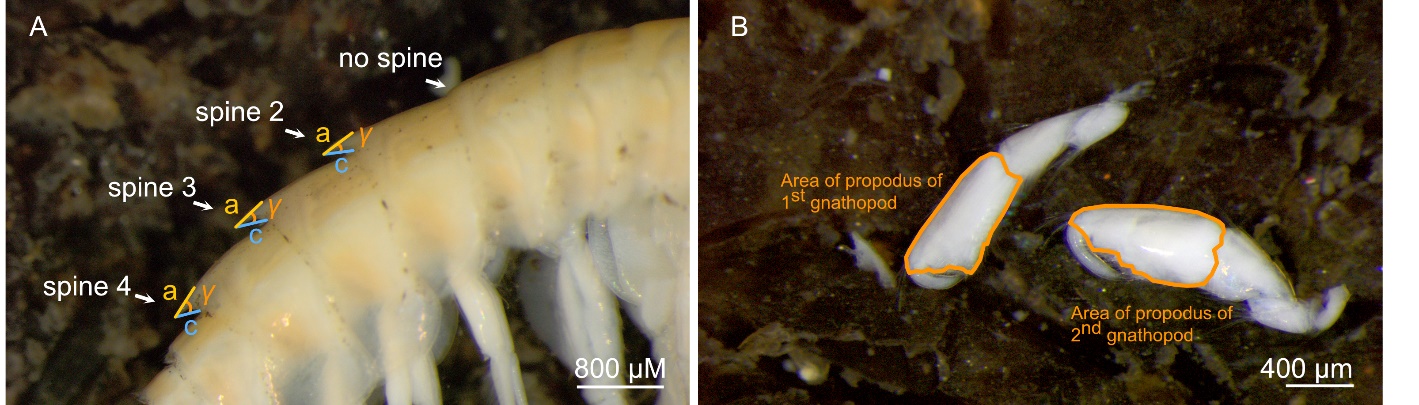


**Figure S1 Spine length and gnathopod area measurement.** A) The angle of each spine as well as the lengths of the inner and outer curve was measured and the area was then calculated. B) The area of the propodus of the gnathopod was measured by circling the area.

**Table S3 Body-length corrected morphological and life-history traits per MOTU**

|  | **MOTU** | **A** | **C** | **G** | **K** | **L** |
| --- | --- | --- | --- | --- | --- | --- |
| **Body length**  **[mm]** | **Mean (± sd)** | 9.83 (± 1.753) | 11.00 (± 2.348) | 10.68 (± 2.030) | 10.62 (± 2.234) | 10.98 (± 1.99) |
|  | **Max** | 17.05 | 20.25 | 18.23 | 17.50 | 15.76 |
|  | **Min** | 6.39 | 5.12 | 6.46 | 5.01 | 6.37 |
| **Spine area**  **[mm²]** | **Mean (± sd)** | 0.08 (± 0.03) | 0.10 (± 0.05) | 0.10 (± 0.04) | 0.10 (± 0.04) | 0.10 ( 0.04) |
|  | **Max** | 0.24 | 0.33 | 0.27 | 0.25 | 0.21 |
|  | **Min** | 0.02 | 0.00 | 0.02 | 0.00 | 0.02 |
| **1^st^ Antenna [mm]** | **Mean (± sd)** | 4.49 (± 0.74) | 4.96 (± 0.95) | 4.84 (± 0.83) | 4.81 (± 0.92) | 4.97 (± 0.84) |
|  | **Max** | 7.10 | 7.83 | 7.37 | 7.20 | 6.77 |
|  | **Min** | 2.89 | 2.36 | 3.06 | 2.56 | 2.88 |
| **2^nd^ Antenna [mm]** | **Mean (± sd)** | 2.48 (± 0.59) | 2.77 (0.74) | 2.73 ( 0.68) | 2.70 (± 0.72) | 2.78 (± 0.67) |
|  | **Max** | 4.50 | 4.94 | 4.66 | 4.56 | 4.32 |
|  | **Min** | 1.51 | 1.17 | 1.60 | 1.20 | 1.50 |
| **Gill area [mm²]** | **Mean (± sd)** | 4.50 (± 1.58) | 5.58 (± 2.19) | 5.27 (± 1.88) | 5.23 (± 2.05) | 5.55 (± 1.85) |
|  | **Max** | 11.54 | 14.96 | 12.80 | 12.02 | 10.18 |
|  | **Min** | 1.61 | 0.69 | 1.74 | 0.79 | 1.59 |
| **1^st^ gnathopod**  **[mm²]** | **Mean (± sd)** | 0.34 (± 0.13) | 0.41 (± 0.18) | 0.40 (± 0.14) | 0.40 (± 0.16) | 0.41 (± 0.15) |
|  | **Max** | 0.75 | 0.89 | 0.80 | 0.77 | 0.69 |
|  | **Min** | 0.07 | 0.00 | 0.10 | 0.06 | 0.07 |
| **2nd gnathopod [mm²]** | **Mean (± sd)** | 0.31 (± 0.11) | 0.38 (± 0.16) | 0.37 (± 0.12) | 0.36 (± 0.14) | 0.37 (± 0.13) |
|  | **Max** | 0.70 | 0.87 | 0.76 | 0.72 | 0.62 |
|  | **Min** | 0.07 | 0.00 | 0.10 | 0.06 | 0.07 |
| **Number of eggs** | **Mean (± sd)** | 13.82 (± 4.94) | 18.66 (± 4.80) | 17.36 (± 6.88) | 17.38 (± 5.00) | 17.92 (± 6.73) |
|  | **Max** | 27.00 | 34.00 | 56.00 | 37.00 | 45.00 |
|  | **Min** | 5.00 | 9.00 | 8.00 | 8.00 | 9.00 |
| **Egg volume [mm³]** | **Mean (± sd)** | 0.09 (± 0.03) | 0.08 (± 0.02) | 0.08 (± 0.01) | 0.08 (± 0.01) | 0.08 (± 0.02) |
|  | **Max** | 0.21 | 0.14 | 0.14 | 0.13 | 0.19 |
|  | **Min** | 0.07 | 0.07 | 0.07 | 0.07 | 0.07 |
| **Egg stage** | **Mean (± sd)** | 2.50 (± 0.93) | 2.41 (± 0.53) | 2.23 (± 0.53) | 2.10 (± 0.34) | 2.27 (± 0.73) |
|  | **Max** | 7.49 | 4.40 | 4.12 | 3.02 | 5.36 |
|  | **Min** | 1.46 | 1.86 | 1.47 | 1.56 | 1.55 |

**Table S4 Climatic data present at each sampling site with data obtained from** [**Domisch et al. (2015)**](#_ENREF_1)**.** The corresponding units are displayed in brackets.

| **Site** | **Flow length [count of grid cells]** | **Cultivated land cover "lc_avg_07", [% cover]** | **Urban land cover "lc_avg_09", [% cover]** | **Annual mean temperature "Bioclim1", [°C]** | **Annual precipitation "Bioclim12", [mm]** |
| --- | --- | --- | --- | --- | --- |
| **1** | 20 | 65 | 0 | 8.25 | 1509 |
| **2** | 125 | 52 | 0 | 8.6 | 1412 |
| **3** | 20494 | 19 | 1 | 6.4 | 2334 |
| **4** | 22637 | 14 | 1 | 6.5 | 2316 |
| **5** | 17844 | 31 | 2 | 6.5 | 2068 |
| **6** | 18456 | 37 | 1 | 6.6 | 2016 |
| **7** | 368 | 64 | 0 | 9.8 | 1784 |
| **9** | 24730 | 31 | 2 | 6.8 | 2208 |
| **10** | 20445 | 62 | 1 | 10.8 | 2414 |
| **11** | 802 | 73 | 1 | 11.2 | 1940 |
| **12** | 49 | 73 | 5 | 11.9 | 1858 |
| **13** | 358 | 29 | 1 | 11.1 | 1730 |
| **15** | 358 | 29 | 1 | 11.1 | 1730 |
| **16** | 65 | 91 | 3 | 10.4 | 1772 |
| **18** | 27 | 56 | 0 | 10.1 | 1818 |
| **19** | 2722 | 63 | 1 | 9.9 | 1638 |
| **20** | 51 | 83 | 0 | 10.1 | 1694 |
| **21** | 1383 | 41 | 0 | 9.9 | 1558 |
| **23** | 315 | 61 | 1 | 9.9 | 1246 |
| **24** | 225 | 60 | 2 | 9.4 | 1278 |
| **25** | 365 | 53 | 2 | 11.3 | 1164 |
| **26** | 1818 | 56 | 2 | 11.2 | 1162 |
| **27** | 248 | 48 | 2 | 11 | 1392 |
| **28** | 1851 | 66 | 2 | 10.2 | 1552 |
| **29** | 3111 | 65 | 1 | 10.6 | 1480 |
| **30** | 459 | 70 | 4 | 14.9 | 1570 |
| **31** | 56 | 63 | 0 | 15.6 | 1602 |
| **33** | 3310 | 64 | 3 | 14.3 | 1504 |
| **34** | 770 | 33 | 0 | 14.7 | 1518 |
| **35** | 332 | 45 | 1 | 14.7 | 1460 |
| **37** | 714 | 48 | 1 | 15.3 | 1572 |
| **38** | 1578 | 43 | 1 | 15.4 | 1550 |
| **40** | 2148 | 42 | 2 | 15.7 | 1504 |
| **41** | 303 | 56 | 1 | 14.7 | 1456 |
| **42** | 16093 | 60 | 2 | 15 | 1168 |
|  | | | | | |
| **Min** | 20.0 | 14.0 | 0.0 | 6.4 | 1162.0 |
| **Max** | 24730.0 | 91.0 | 5.0 | 15.7 | 2414.0 |
| **Mean** | 4703.7 | 52.7 | 1.3 | 11.1 | 1656.5 |
| **sd** | 7842.0 | 17.3 | 1.1 | 2.9 | 324.0 |

**Table S5 Environmental parameters and results from the in vitro assays at each sampling site.** The corresponding units are displayed in brackets.

| **Site** | **Altitude [m]** | **pH** | **Conductivity [µS/cm]** | **O_2_ saturation [%]** | **Temperature [°C]** | **Nitrate [mg/L]** | **Nitrite [mg/L]** | **Phosphate [mg/L]** | **Carbonate hardness [°dH]** | **YES [ng/g]** | **YDS [mg/g]** | **EC_50_-100 [mg sediment equivalency]** |
| --- | --- | --- | --- | --- | --- | --- | --- | --- | --- | --- | --- | --- |
| 1 | 400 | 9.76 | 225 | 100.1 | 14 | 28 | 0.00 | 0.24 | 1.60 | 0.000 | 1.11 | 88.68 |
| 2 | 223 | 8.19 | 510 | 105.2 | 14 | 12 | 0.00 | 0.12 | 14.40 | 0.000 | 4.07 | 65.73 |
| 3 | 343 | 7.53 | 253 | 96.8 | 15.7 | 0 | 0.00 | 0.00 | 6.00 | 0.000 | 0.17 | 7.38 |
| 4 | 247 | 7.63 | 255 | 101 | 17.3 | 0 | 0.00 | 0.00 | 6.20 | 0.654 | 4.60 | 95.31 |
| 5 | 184 | 7.8 | 313 | 108.9 | 15.3 | 0 | 0.00 | 0.02 | 6.60 | 0.629 | 2.13 | 83.40 |
| 6 | 170 | 7.77 | 441 | 116.5 | 20.2 | 0 | 0.00 | 0.00 | 11.00 | 1.102 | 0.29 | 96.17 |
| 7 | 178 | 7.88 | 414 | 118.2 | 21.9 | 1 | 0.05 | 0.00 | 15.40 | 1.221 | 0.19 | 97.78 |
| 9 | 211 | 7.95 | 270 | 107.4 | 17 | 2 | 0.02 | 0.14 | 6.40 | 7.916 | 3.32 | 96.70 |
| 10 | 12 | 7.74 | 278 | 104 | 24.4 | 0 | 0.00 | 0.27 | 7.20 | 0.000 | 0.49 | 87.28 |
| 11 | 411 | 8.54 | 565 | 135 | 20.8 | 0 | 0.01 | 0.19 | 18.20 | 0.000 | 0.21 | 72.17 |
| 12 | 578 | 8.01 | 486 | 100.9 | 20.4 | 5 | 0.06 | 0.32 | 16.20 | 0.000 | 0.44 | 49.27 |
| 13 | 692 | 8.21 | 216.2 | 136.2 | 23.4 | 0 | 0.02 | 0.05 | 5.40 | 3.229 | 6.97 | 96.60 |
| 15 | 696 | 8.17 | 222 | 125.4 | 22.8 | 0 | 0.01 | 0.22 | 6.20 | 0.000 | 0.80 | 94.57 |
| 16 | 832 | 8.08 | 500 | 118.2 | 17.1 | 11 | 0.25 | 0.40 | 14.20 | 2.862 | 2.01 | 97.71 |
| 18 | 905 | 8.25 | 473 | 101.8 | 17.4 | 0 | 0.01 | 0.50 | 15.60 | 0.392 | 0.45 | 95.44 |
| 19 | 826 | 8.19 | 563 | 130.3 | 19.7 | 0 | 0.04 | 0.15 | 18.20 | 0.000 | 0.00 | 96.24 |
| 20 | 847 | 8.6 | 210.6 | 162 | 24 | 0 | 0.00 | 0.00 | 5.60 | 0.769 | 0.33 | 95.85 |
| 21 | 851 | 8.42 | 254 | 139.3 | 19.3 | 0 | 0.00 | 0.10 | 7.40 | 0.000 | 1.69 | 53.68 |
| 23 | 628 | 8.07 | 377 | 99.5 | 19.6 | 0 | 0.06 | 0.16 | 7.60 | 5.628 | 2.83 | 96.35 |
| 24 | 668 | 8.39 | 342 | 105.8 | 17.1 | 0 | 0.02 | 0.08 | 5.60 | 1.076 | 0.47 | 74.57 |
| 25 | 574 | 8.67 | 1100 | 179.9 | 21.4 | 0 | 0.00 | 0.01 | 19.00 | 0.000 | 0.17 | 89.33 |
| 26 | 517 | 8.85 | 639 | 159 | 22.1 | 0 | 0.01 | 0.03 | 11.00 | 0.427 | 0.00 | 72.58 |
| 27 | 628 | 8.66 | 292 | 104.7 | 21.3 | 0 | 0.02 | 0.00 | 4.80 | 4.819 | 17.90 | 97.49 |
| 28 | 613 | 8.19 | 452 | 97.1 | 16 | 12 | 0.12 | 0.21 | 12.60 | 1.220 | 0.69 | 98.61 |
| 29 | 554 | 8.3 | 495 | 126.6 | 19.1 | 9 | 0.03 | 0.32 | 12.00 | 0.457 | 0.14 | 77.00 |
| 30 | 114 | 7.91 | 489 | 81.8 | 19.8 | 5 | 0.05 | 0.20 | 14.40 | 0.342 | 0.24 | 91.65 |
| 31 | 129 | 8.17 | 368 | 112.1 | 18.7 | 0 | 0.00 | 0.18 | 11.00 | 0.395 | 0.55 | 0.00 |
| 33 | 92 | 7.96 | 590 | 70.8 | 22.3 | 20 | 0.14 | 1.61 | 15.60 | 2.194 | 0.00 | 96.77 |
| 34 | 183 | 8.49 | 461 | 126.2 | 14.9 | 0 | 0.03 | 0.05 | 12.60 | 0.000 | 0.65 | 35.44 |
| 35 | 392 | 9.01 | 697 | 170.7 | 21.4 | 20 | 0.51 | 0.17 | 21.60 | 0.000 | 0.87 | 98.75 |
| 37 | 100 | 7.85 | 497 | 117.4 | 22.3 | 0 | 0.02 | 0.02 | 13.20 | 0.000 | 0.22 | 75.00 |
| 38 | 33 | 8.02 | 564 | 98.7 | 18.5 | 5 | 0.01 | 0.04 | 14.60 | 0.000 | 0.00 | 97.08 |
| 40 | 7 | 8.09 | 467 | 94.2 | 19.7 | 2 | 0.05 | 0.11 | 13.80 | 0.000 | 0.23 | 78.50 |
| 41 | 463 | 8.27 | 823 | 158 | 19.8 | 100 | 1.64 | 0.13 | 25.40 | 0.000 | 0.34 | 64.20 |
| 42 | 13 | 7.73 | 729 | 102.3 | 19.1 | 0 | 0.01 | 0.50 | 18.20 | 0.000 | 0.27 | 41.93 |
|  | | | | | | | | | | | | |
| **Min** | 7 | 7.53 | 211 | 70.8 | 14.0 | 0 | 0.00 | 0.00 | 1.60 | 0.000 | 0.00 | 0.00 |
| **Max** | 905 | 9.76 | 1100 | 179.9 | 24.4 | 100 | 1.64 | 1.61 | 25.40 | 7.916 | 17.90 | 98.75 |
| **Mean** | 409 | 8.21 | 452 | 117.5 | 19.4 | 7 | 0.09 | 0.19 | 11.85 | 1.009 | 1.57 | 78.72 |
| **sd** | 280 | 0.43 | 190 | 24.7 | 2.8 | 17 | 0.28 | 0.28 | 5.44 | 1.796 | 3.19 | 25.17 |


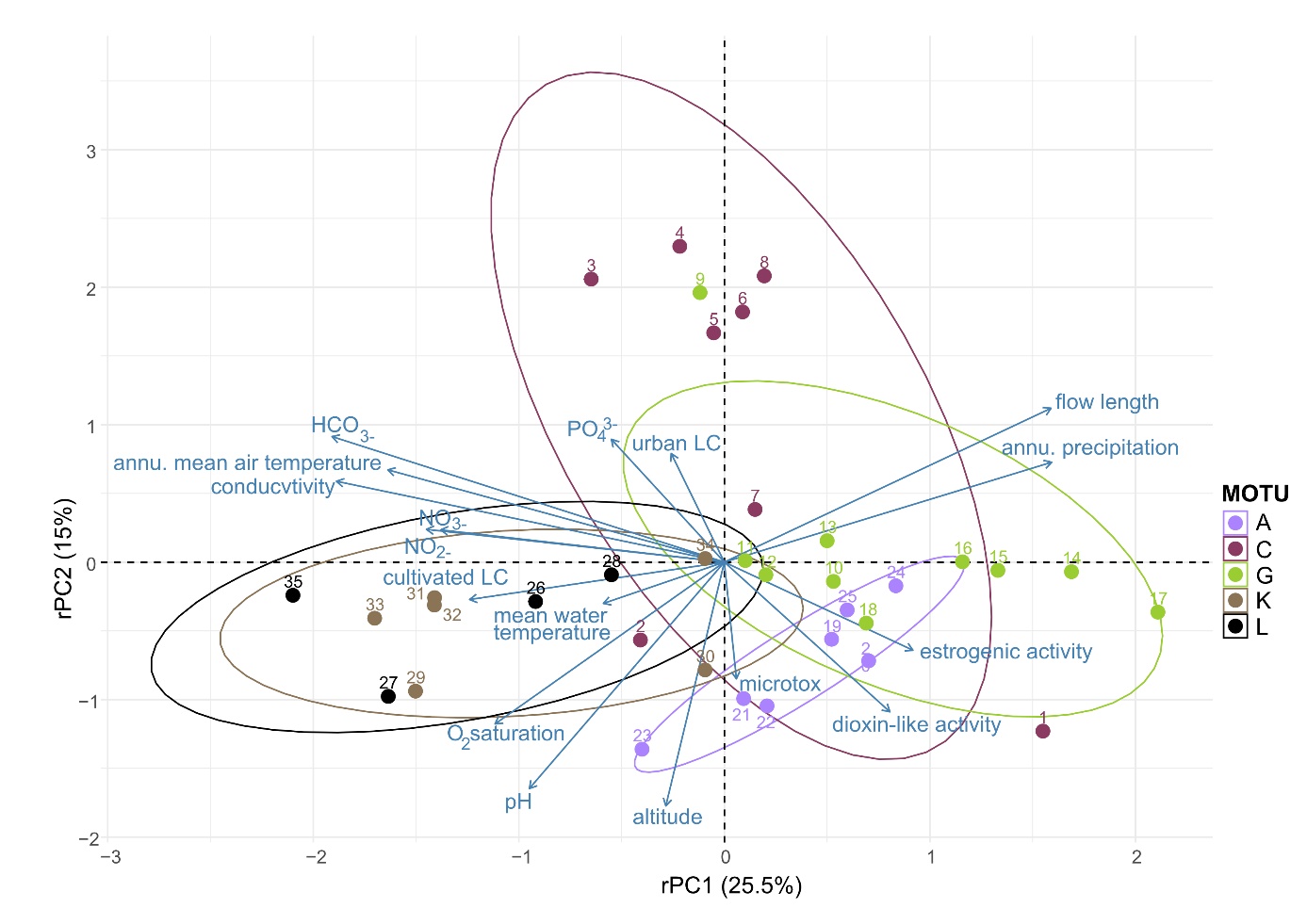


**Figure S2 Varimax rotated PCA with all environmental parameters considered.** rPC1 explains 25.5 % while rPC2 explains15.0 % of the overall variance in the dataset. The colours represent the respective MOTUs. The numbers represent the sampling sites.

**Table S6 Loadings or the rotated PCA with their respective variance and significance.** Only the first two axes were significant for the anaylsis. The parameters influencing the PCA with over 0.6 are highlighted with a gray.

|  | **rPC1** | **rPC2** | **rPC3** | **rPC4** |
| --- | --- | --- | --- | --- |
| **Altitude** | -0.119 | -0.740 | -0.082 | 0.077 |
| **pH** | -0.396 | -0.687 | 0.098 | -0.129 |
| **Conductivity** | -0.787 | 0.245 | 0.020 | 0.046 |
| **O2 saturation** | -0.465 | -0.491 | 0.462 | 0.078 |
| **Mean water temperature** | -0.245 | -0.125 | -0.287 | -0.006 |
| **Nitrate** | -0.576 | 0.098 | 0.229 | 0.650 |
| **Nitrite** | -0.605 | 0.099 | 0.258 | 0.695 |
| **Phosphate** | -0.229 | 0.373 | -0.594 | 0.021 |
| **Carbonate hardness** | -0.796 | 0.381 | 0.041 | 0.111 |
| **YES** | 0.383 | -0.267 | -0.492 | 0.471 |
| **YDS** | 0.336 | -0.454 | -0.254 | 0.337 |
| **Flow length** | 0.663 | 0.467 | 0.190 | 0.298 |
| **Annu. Precipitation** | 0.664 | 0.303 | 0.181 | 0.250 |
| **Cultivated land cover** | -0.517 | -0.113 | -0.459 | -0.192 |
| **Urban land cover** | -0.108 | 0.330 | -0.635 | 0.143 |
| **annu. Mean temperature** | -0.683 | 0.280 | -0.156 | -0.244 |
| **EC_50_-100** | 0.025 | -0.354 | -0.455 | 0.352 |
|  | | | | |
| **Variance [%]** | 25.51 | 15.02 | 11.81 | 9.86 |
| **Cumulative variance [%]** | 25.51 | 40.53 | 52.34 | 62.2 |
| **p-value** | < 0.001 | < 0.001 | 0.07 | 0.24 |

**Table S7 Loadings of PCA for phenotypic traits used in Discriminant Analysis.**

|  | **PC1** | **PC2** | **PC3** | **PC4** | **PC5** | **PC6** |
| --- | --- | --- | --- | --- | --- | --- |
| **Male** | | | | | | |
| **Standard Deviation** | 1.612 | 1.214 | 1.003 | 0.905 | 0.798 | 0.577 |
| **Proportion of Variance** | 0.371 | 0.210 | 0.144 | 0.117 | 0.091 | 0.048 |
| **Cumulative Proportion** | 0.371 | 0.582 | 0.725 | 0.842 | 0.933 | 0.981 |
| **Female** | | | | | | |
| **Standard Deviation** | 1.373 | 1.112 | 1.037 | 1.008 | 0.858 | 0.833 |
| **Proportion of Variance** | 0.269 | 0.177 | 0.154 | 0.145 | 0.105 | 0.099 |
| **Cumulative Proportion** | 0.269 | 0.446 | 0.600 | 0.745 | 0.850 | 0.949 |

**Table S8 Discriminant analysis Eigenvalues for each Discriminant Function retained and explained variance in percent.**

|  | **LD1** | **LD2** | **LD3** | **LD4** |
| --- | --- | --- | --- | --- |
| **Male** | | | | |
| **DA Eigenvalue** | 51.72 | 41.56 | 6.17 | 1.43 |
| **Explained variance [%]** | 51.27 | 41.20 | 6.11 | 1.42 |
| **Female** | | | | |
| **DA Eigenvalue** | 50.28 | 26.02 | 4.80 | 2.68 |
| **Explained variance [%]** | 60.02 | 31.06 | 5.73 | 3.19 |

**Table S9 Group discrimination by the discriminant function for male and female phenotypic traits.**

|  | **A** | **C** | **G** | **K** | **L** | **Overall** |
| --- | --- | --- | --- | --- | --- | --- |
| **Males** | 0.7426 | 0.4467 | 0.6414 | 0.0446 | 0.4675 | 0.4993 |
| **Females** | 0.8488 | 0.4769 | 0.4500 | 0.1111 | 0.7083 | 0.5651 |

**Table S10 Phenotypic variable contribution to the discriminant function for male and female traits.**

| **Males** | | | | |
| --- | --- | --- | --- | --- |
| **Variable** | **LD1** | **LD2** | **LD3** | **LD4** |
| **Body length** | 0.0005 | 0.0000 | 0.0000 | 0.0001 |
| **Spine area** | 0.2623 | 0.9588 | 0.9540 | 0.0000 |
| **1^st^ Antenna** | 0.0638 | 0.0007 | 0.0003 | 0.0002 |
| **2^nd^ Antenna** | 0.0452 | 0.0003 | 0.0114 | 0.0003 |
| **Gill area** | 0.0031 | 0.0003 | 0.0080 | 0.0000 |
| **1^st^ Gnathopod** | 0.5963 | 0.0360 | 0.0259 | 0.6222 |
| **2^nd^ Gnathopod** | 0.0287 | 0.0039 | 0.0004 | 0.3771 |
| **Females** | | | | |
| **Variable** | **LD1** | **LD2** | **LD3** | **LD4** |
| **Body length** | 0.0011 | 0.0000 | 0.0000 | 0.0001 |
| **Spine area** | 0.8847 | 0.8699 | 0.0106 | 0.8752 |
| **1^st^ Antenna** | 0.0010 | 0.0117 | 0.0001 | 0.0000 |
| **2^nd^ Antenna** | 0.0005 | 0.0100 | 0.0060 | 0.0057 |
| **Gill area** | 0.0002 | 0.0010 | 0.0000 | 0.0004 |
| **Fecundity** | 0.0000 | 0.0000 | 0.0000 | 0.0000 |
| **Egg volume** | 0.1126 | 0.1075 | 0.9833 | 0.1187 |

# References

Domisch, S., Amatulli, G. & Jetz, W. 2015. Near-global freshwater-specific environmental variables for biodiversity analyses in 1 km resolution. *Scientific Data,* 2**,** 1-13
